# Feasibility and Preliminary Effects of AI-Generated Personalized Sleep Feedback in High School Female Soccer Players: Pilot Randomized Controlled Trial

**DOI:** 10.2196/101615

**Published:** 2026-10-01

**Authors:** Hayato Kedoin, Yuzuru Itoh, Takumi Nirengi, Yuji Sato, Shun Sugisawa, Yusuke Nishio, Ryo Akitsu

**Affiliations:** 1 Doctoral Program in Judo Therapy Graduate School of Medical and Health Science Nippon Sport Science University Yokohama, Kanagawa Japan; 2 Nippon Sport Science University Medical Vocational School Tokyo Japan; 3 Division of Sport Cure Center Nippon Sport Science University Yokohama, Kanagawa Japan; 4 Department of Judo Therapy and Medical Science Faculty of Medical Science Nippon Sport Science University Yokohama, Kanagawa Japan; 5 Department of Judo Therapy Faculty of Medical Technology Teikyo University Utsunomiya, Tochigi Japan; 6 Department of Judo Physical Therapy Faculty of Health Care and Medical Sports Teikyo Heisei University Tokyo Japan; 7 Master’s Program in Advanced Practical Judo Therapy Graduate School of Medical and Health Science Nippon Sport Science University Yokohama, Kanagawa Japan

**Keywords:** adolescent athletes, AI, artificial intelligence, female soccer players, generative AI, mHealth, mobile health, personalized feedback, pilot randomized controlled trial, sleep, wearable devices

## Abstract

**Background:**

Adequate sleep supports mood regulation and injury prevention in high school female athletes; however, insufficient sleep is common in this population. Wearable devices enable continuous assessment of objective sleep metrics, but personalized feedback based on daily sleep data remains underexplored.

**Objective:**

This study evaluated the technical and operational feasibility of a multicomponent AI-supported mobile health (mHealth) intervention that delivered personalized sleep feedback to high school female soccer players. Sleep efficiency was the primary preliminary efficacy outcome; other sleep-related measures were secondary or exploratory outcomes, and mood state and sports injury severity were exploratory outcomes.

**Methods:**

We conducted a pilot randomized controlled trial among 25 high school female soccer players from a single team. The study briefing and system setup began on August 18, 2025, and the trial was retrospectively registered with the University Hospital Medical Information Network (UMIN) Clinical Trials Registry on April 7, 2026 (UMIN000061189). Weeks 1 to 4 constituted the baseline period. After the week 5 baseline questionnaires, participants were stratified by median baseline total sleep time (TST) and Pittsburgh Sleep Quality Index score and randomly allocated 1:1 to the intervention (n=12) or control (n=13) group. During weeks 6 to 9, the intervention group received GPT-4o–generated personalized feedback via LINE on days with valid prior-night Fitbit data, whereas the control group received daily nonpersonalized sleep information. Technical and operational feasibility outcomes included message delivery success, valid Fitbit wear nights, questionnaire completion, technical problems, adverse events, and privacy breaches. Secondary outcomes included TST, wake after sleep onset (WASO), subjective sleep quality, sleep difficulty, and daytime sleepiness. Deep, light, and rapid eye movement (REM) sleep durations, mood state, and sports injury severity were exploratory outcomes.

**Results:**

All 25 participants completed the trial. Message delivery success and questionnaire completion were 100% in both groups. Median valid Fitbit wear nights were 26 (IQR 24-28) in the intervention group and 22 (IQR 15-26) in the control group. No major technical problems, adverse events, or privacy breaches occurred. Change in sleep efficiency, the primary preliminary efficacy outcome, favored the intervention group (Hodges-Lehmann between-group difference 2%, 95% CI 0.50%-4%; *P*=.046; *r*=0.35). Nominal between-group differences were also observed for the Japanese version of the Pittsburgh Sleep Quality Index (PSQI-J) total score and sleep difficulty score and, among exploratory outcomes, REM sleep duration and total mood disturbance. No between-group differences were observed in TST, WASO, deep or light sleep duration, daytime sleepiness, or sports injury severity.

**Conclusions:**

The multicomponent AI-supported mHealth intervention demonstrated technical and operational feasibility in this single-team setting. Preliminary, hypothesis-generating between-group differences were observed in sleep efficiency and selected subjective sleep and mood outcomes. These findings should not be interpreted as evidence of established effectiveness and require confirmation in a sufficiently powered trial.

**Trial Registration:**

UMIN Clinical Trials Registry UMIN000061189; https://tinyurl.com/tw3d7aku

## Introduction

Adequate sleep is essential for athletes’ physical recovery, performance, and injury prevention [1]. Sleep supports athletic conditioning through physiological and cognitive processes, including growth hormone secretion, which facilitates skeletal muscle repair, and memory consolidation, which underpins the acquisition and retention of motor skills [2]. These recovery processes are particularly critical for high school athletes, who are still undergoing physical and psychological development. However, high school athletes are required to balance sport participation with academic demands [3], and constraints such as training schedules, commuting, and schoolwork can disrupt sleep and limit adequate recovery [4]. Insufficient sleep is associated with not only delayed reaction time and reduced cognitive function, including impaired judgment, but also increased anxiety and worsened mood [5]. Chronic sleep insufficiency may further impair neuromuscular control and increase the risk of sports injuries [6,7]. Therefore, effective conditioning strategies are needed to help high school athletes optimize sleep within their limited available time.

Various interventions have been implemented to address sleep-related problems in athletes, including sleep education, sleep hygiene guidance, sleep extension, and behavior change strategies [8,9]. Sleep education may improve sleep awareness and contribute to increased sleep duration, reduced sleep onset latency, and improved sleep efficiency [10]. The increasing availability of wearable devices has facilitated the objective assessment of sleep in daily life [11,12], and mobile interventions that provide feedback based on these data have emerged as a promising approach to improving sleep behaviors [13,14]. Nevertheless, many existing interventions rely on group-based instruction, standardized information delivery, or self-monitoring. Approaches for translating day-to-day objective sleep data into individualized, actionable guidance remain insufficiently developed. Effective use of objective data for behavior change also requires mobile tools that are acceptable to athletes and capable of delivering timely support. In this context, communication applications such as LINE (LY Corporation), which is widely used among high school students in Japan, may enable continuous and personalized support in sports settings [15].

In recent years, generative AI based on large language models has garnered attention as a potential solution for delivering personalized interventions [16]. Because generative AI can produce natural-language responses tailored to an individual’s condition and context, it may be useful for delivering personalized health information and supporting behavior change [16,17]. More broadly, in health care contexts, AI chatbot interventions have demonstrated potential to improve health behaviors [17], and in the field of sleep, chatbot-based interventions in adults have been shown to improve sleep habits and reduce insomnia symptoms [18]. However, few studies have linked objective sleep data obtained from wearable devices with personalized feedback generated by generative AI in athletic populations, and the feasibility and preliminary effects of such approaches remain unclear. This gap is particularly relevant for high school female athletes, who often face demanding schedules that include early-morning practices, prolonged after-school training sessions, and travel for competitions. Interventions that automatically generate and deliver individualized morning feedback based on objective sleep data from the previous night have not been sufficiently evaluated in this population. Moreover, female athletes may be at increased risk of injury due to anatomical and physiological factors [19], suggesting that improvements in sleep behavior may be relevant not only for mood state but also for injury prevention.

This study focused on high school female soccer players for several reasons. First, achieving sufficient sleep is particularly challenging during high school, when students must balance academic responsibilities with club activities, making sustainable sleep support strategies in sports settings practically important. Second, soccer involves training, matches, and travel, which can lead to fluctuations in daily routines that influence sleep and mood state. Third, focusing on a single team allowed us to examine technical and operational feasibility and preliminary effects while partially controlling environmental factors such as training schedules and coaching structure. Despite the relevance of this population, most previous studies have focused on adults or university students and have relied on group-based education, general information provision, or self-monitoring. Few studies have evaluated interventions that automatically generate and deliver individualized morning messages to adolescent female athletes based on objective sleep data collected via wearable devices during the preceding night. Moreover, few studies have simultaneously assessed mood state and sports injury severity alongside sleep outcomes. This study addresses these gaps by using objective sleep data collected in a real-world sports setting to generate personalized feedback via generative AI and by examining sleep, mood state, and injury-related outcomes concurrently.

Accordingly, we aimed to evaluate the technical and operational feasibility of a mobile health (mHealth) intervention that automatically delivered personalized feedback generated by generative AI via LINE, based on objective sleep metrics obtained from Fitbit data on nights with valid recordings, among high school female soccer players. We also examined preliminary changes in sleep-related outcomes, with sleep efficiency designated as the primary preliminary efficacy outcome for this pilot report. Other sleep-related measures were evaluated as secondary or exploratory outcomes. We hypothesized that the intervention group would show a greater change in sleep efficiency than the control group. Mood state and sports injury severity were assessed as exploratory outcomes.

## Methods

### Study Design

This exploratory pilot trial used a parallel-group, stratified randomized controlled design with preintervention and postintervention comparisons between an intervention group and a control group. Prior to conducting a full-scale effectiveness trial, this pilot study aimed to assess the technical and operational feasibility of the intervention system and to examine preliminary changes in sleep-related outcomes. Sleep efficiency was designated as the primary preliminary efficacy outcome for this pilot report. Other outcomes were classified as secondary or exploratory outcomes, as described below.

This study was reported in accordance with the CONSORT (Consolidated Standards of Reporting Trials) 2010 statement and the CONSORT-EHEALTH (Consolidated Standards of Reporting Trials of Electronic and Mobile Health Applications and Online Telehealth) checklist. The completed CONSORT-EHEALTH checklist is provided in Multimedia Appendix 1. Because this study evaluated an AI-supported behavioral feedback system rather than an autonomous diagnostic or treatment decision system, AI-related reporting elements were addressed where applicable.

### Study Flow

The study flow is shown in Figure 1. The study briefing and initial setup were conducted on August 18, 2025; baseline observation began on September 1, 2025; and the final assessment was completed on November 3, 2025.

At study initiation (week 0), a briefing session was held for players, their parents or guardians, and coaching staff. Participants provided assent, and written informed consent was obtained from their parents or guardians. During this session, wearable devices (Fitbit Inspire 3; Google LLC) were distributed, required apps were configured, and participants were enrolled in the study’s LINE official account for study-related communication.

During the baseline observation period (weeks 1-4), participants continuously recorded sleep metrics using Fitbit devices, including total sleep time (TST), wake after sleep onset (WASO), sleep efficiency, and sleep-stage variables. No intervention was delivered during this period, and participants did not receive personalized feedback or behavioral recommendations generated by generative AI.

In week 5, participants completed baseline questionnaires. They were then stratified based on data from the baseline observation period (weeks 1-4) and randomly allocated in a 1:1 ratio to either the intervention or control group.

During the intervention period (weeks 6-9), Fitbit-based sleep monitoring continued in both groups, and study messages were delivered via LINE according to each participant’s assigned condition. When valid previous-night Fitbit data were available, the intervention group received personalized feedback generated by GPT-4o (OpenAI) based on these objective sleep metrics. When valid sleep data were unavailable, no personalized feedback was generated, and no intervention message was delivered for that day. The control group received standardized, nonpersonalized sleep information each morning at 8 AM that did not incorporate individual data. Both groups received sleep-related messages via LINE; however, the intervention and control conditions differed in personalization, use of prior-night and baseline data, message-generation procedures, delivery timing, behavioral specificity, and the number of messages received because intervention messages were sent only on days with valid prior-night data. Therefore, the comparison did not isolate the effect of any single intervention component. Postintervention questionnaires were completed in week 10.

**Figure 1:**
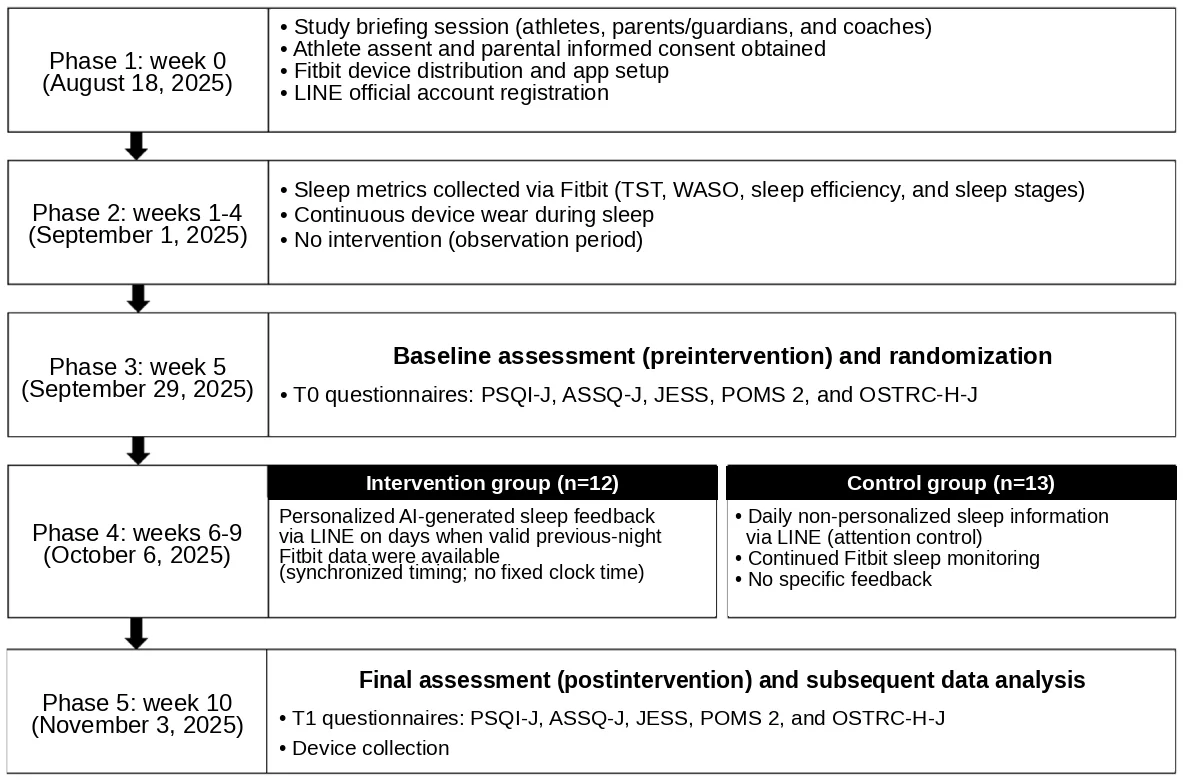
Study timeline and procedures for the pilot randomized controlled trial. Week 0 included the study briefing, informed consent procedures, device distribution, app setup, and registration for the LINE official account. Weeks 1 to 4 comprised the baseline observation period, during which participants underwent continuous Fitbit-based sleep monitoring. In week 5, participants completed baseline questionnaires and underwent randomization. During weeks 6 to 9, the intervention group received AI-generated personalized sleep feedback via LINE on days with valid Fitbit sleep data from the previous night, whereas the control group received daily nonpersonalized sleep information. In week 10, participants completed postintervention questionnaires, and researchers collected the devices. ASSQ-J: Japanese version of the Athlete Sleep Screening Questionnaire; JESS: Japanese version of the Epworth Sleepiness Scale; OSTRC-H-J: Japanese version of the Oslo Sports Trauma Research Center Questionnaire on Health Problems; POMS 2: Profile of Mood States, Second Edition; PSQI-J: Japanese version of the Pittsburgh Sleep Quality Index; TST: total sleep time; WASO: wake after sleep onset.

### Participants

Participants were 25 female soccer players from a single high school team. The mean age was 16.93 (SD 0.70) years, and the mean playing experience was 9.61 (SD 2.14) years. All participants were actively engaged in competitive sport and participated in regular training sessions and matches throughout the study period.

As this exploratory pilot trial aimed to assess technical and operational feasibility and estimate the direction and magnitude of preliminary between-group differences prior to a full-scale effectiveness trial, no formal sample size calculation based on an a priori power analysis was conducted for confirmatory hypothesis testing. The sample size was determined pragmatically by enrolling all eligible players from the team who were available during the study period.

### Eligibility Criteria

Participants were eligible if they (1) belonged to the target high school female soccer team, (2) participated in regular training sessions and matches during the study period, (3) were able to wear a Fitbit device throughout the study period, (4) were able to receive study messages via LINE, and (5) provided assent, with written informed consent obtained from a parent or guardian. In addition, participants were required to have access to a smartphone capable of running LINE and synchronizing Fitbit data.

Participants were excluded if continuation in the study was deemed infeasible or if device wear or data synchronization was substantially impaired. No participants were excluded after eligibility screening.

### Stratification Factors

To reduce between-group imbalance in baseline sleep status, 2 stratification factors were used. The first was Fitbit-measured TST during the baseline period. The median baseline TST was calculated, and participants were classified into below-median and at-or-above-median strata. The second was the Japanese version of the Pittsburgh Sleep Quality Index (PSQI-J) total score during the same period. Using a cutoff of 6, participants were classified as having good sleep quality (≤5) or poor sleep quality (≥6).

### Stratum Formation and Random Allocation

Participants were categorized into 4 strata based on the combination of the 2 stratification factors:

- Mean TST below the median and PSQI-J score ≤5
- Mean TST below the median and PSQI-J score ≥6
- Mean TST at or above the median and PSQI-J score ≤5
- Mean TST at or above the median and PSQI-J score ≥6

Within each stratum, allocation sequences were generated using random numbers in Microsoft Excel (Microsoft Corporation) by an independent third party not otherwise involved in the study. The allocation table was concealed from investigators until intervention initiation. Participants were then randomly assigned within each stratum to the intervention or control group in a 1:1 ratio.

Due to the nature of the intervention, neither participants nor investigators were blinded to group assignment. However, for questionnaire data processing and statistical analysis, groups were masked as Group A and Group B, with group identities unblinded only after completion of analyses.

### Outcomes and Measurements

Sleep efficiency was identified as the principal sleep outcome during the initial study planning stage and was subsequently designated as the primary preliminary efficacy outcome for this pilot report. This designation reflected the study’s planned focus on evaluating between-group changes in sleep efficiency as the main sleep-related outcome. Secondary outcomes included TST, WASO, the PSQI-J total score, the sleep difficulty score (SDS) from the Athlete Sleep Screening Questionnaire, and daytime sleepiness assessed using the Japanese version of the Epworth Sleepiness Scale (JESS). Deep sleep duration, light sleep duration, rapid eye movement (REM) sleep duration, total mood disturbance (TMD), and sports injury severity were treated as exploratory outcomes. Because the trial was registered retrospectively, this outcome hierarchy should not be interpreted as prospectively registered prespecification of the type expected in a confirmatory trial.

In this study, feasibility was defined in technical and operational terms. Feasibility outcomes included LINE message delivery success rate, number of valid Fitbit wear nights, questionnaire completion rate, occurrence of technical problems, occurrence of adverse events, and privacy breaches. Message opening or reading, viewing duration, comprehension of message content, satisfaction, perceived burden, acceptability, and willingness to continue using the system were not assessed; therefore, participant engagement and acceptability could not be evaluated.

### Objective Sleep Metrics

Objective sleep metrics were assessed using a Fitbit Inspire 3, a consumer-grade wrist-worn activity tracker. The variables assessed included TST, WASO, sleep efficiency, and the duration of deep sleep, light sleep, and REM sleep. Fitbit is a consumer wearable device that enables relatively low-cost and convenient sleep measurement. It has been used in studies of sleep among athletes, and its sleep parameters demonstrate acceptable validity for field-based sleep monitoring; however, sleep-stage estimates should be interpreted with caution [11,12]. Participants were instructed to wear the device on the wrist opposite their dominant hand during sleep throughout the study period.

For each participant, objective sleep metrics were calculated as mean values across valid wear nights during the baseline observation period (weeks 1-4) and the intervention period (weeks 6-9). A valid wear night was defined as a night in which the Fitbit recorded a main sleep episode and all sleep variables required for analysis were available. No minimum number of valid nights was specified a priori, and missing data were not imputed. Accordingly, analyses for each sleep metric were based on mean values across available valid wear nights.

### Subjective Sleep Quality

Subjective sleep quality was assessed using the PSQI-J [20]. This questionnaire comprises 18 items assessing sleep quality over the previous month. Each component is scored from 0 to 3, yielding a total score ranging from 0 to 21, with higher scores indicating poorer sleep quality. The outcome variable was the PSQI-J total score. Because the PSQI-J requires recall of sleep over the previous month, the week-5 assessment was intended to primarily reflect the baseline observation period (weeks 1-4), whereas the week-10 assessment was intended to primarily reflect the intervention period (weeks 6-9).

### Sleep Behavior and Daytime Sleepiness

Sleep behavior was assessed using the Japanese version of the Athlete Sleep Screening Questionnaire (ASSQ-J) [21], which evaluates sleep quantity and quality, insomnia symptoms, and behaviors related to circadian rhythm. The outcome variable was the SDS.

Daytime sleepiness was assessed using the JESS [22]. Participants rated their likelihood of dozing in 8 situations on a 4-point scale from 0 (“would never doze”) to 3 (“high chance of dozing”), yielding a total score ranging from 0 to 24. The outcome variable was the JESS total score.

### Mood State

Mood state was assessed using the Japanese version of the Profile of Mood States, Second Edition (POMS 2) [23]. The outcome variable was the TMD score, which reflects overall mood disturbance.

### Sports Injury Severity

Sports injury severity was assessed using a modified Japanese version of the Oslo Sports Trauma Research Center Questionnaire on Health Problems (OSTRC-H-J) [24]. The original OSTRC instrument [25] and updated versions [26] were also consulted. For this study, the recall period was modified from the previous 7 days to the previous month, and the scope was restricted to sports injuries rather than general health problems.

The modified questionnaire comprised 4 items assessing participation in training and matches, reductions in training volume, impact on performance, and symptom severity. Responses were weighted according to the method described by Clarsen et al [25], and a severity score ranging from 0 to 100 was calculated as the sum of weighted item scores. Higher scores indicate a greater impact of sports injury on athletic participation and performance.

Because both the recall period and assessment scope were modified, the resulting score was not assumed to retain the measurement properties of the original OSTRC instrument or the validated OSTRC-H-J. It was therefore treated as an exploratory outcome. To align assessment with preintervention and postintervention comparisons, the recall period was set to the previous month: the week-5 assessment was intended to primarily reflect the baseline observation period, whereas the week-10 assessment was intended to primarily reflect the intervention period.

### Intervention

#### Intervention Group: Personalized Sleep Feedback Generated by Generative AI

Participants in the intervention group received personalized sleep feedback generated by GPT-4o (OpenAI), a large language model, based on objective sleep metrics obtained from a Fitbit device. GPT-4o was selected because it provided stable Japanese-language text generation, reliable adherence to structured prompts, and practical integration with the OpenAI Responses API, which were required for automated daily feedback delivery in this study. Feedback was provided on days with available valid previous-night Fitbit sleep data (Figure 2).

**Figure 2:**
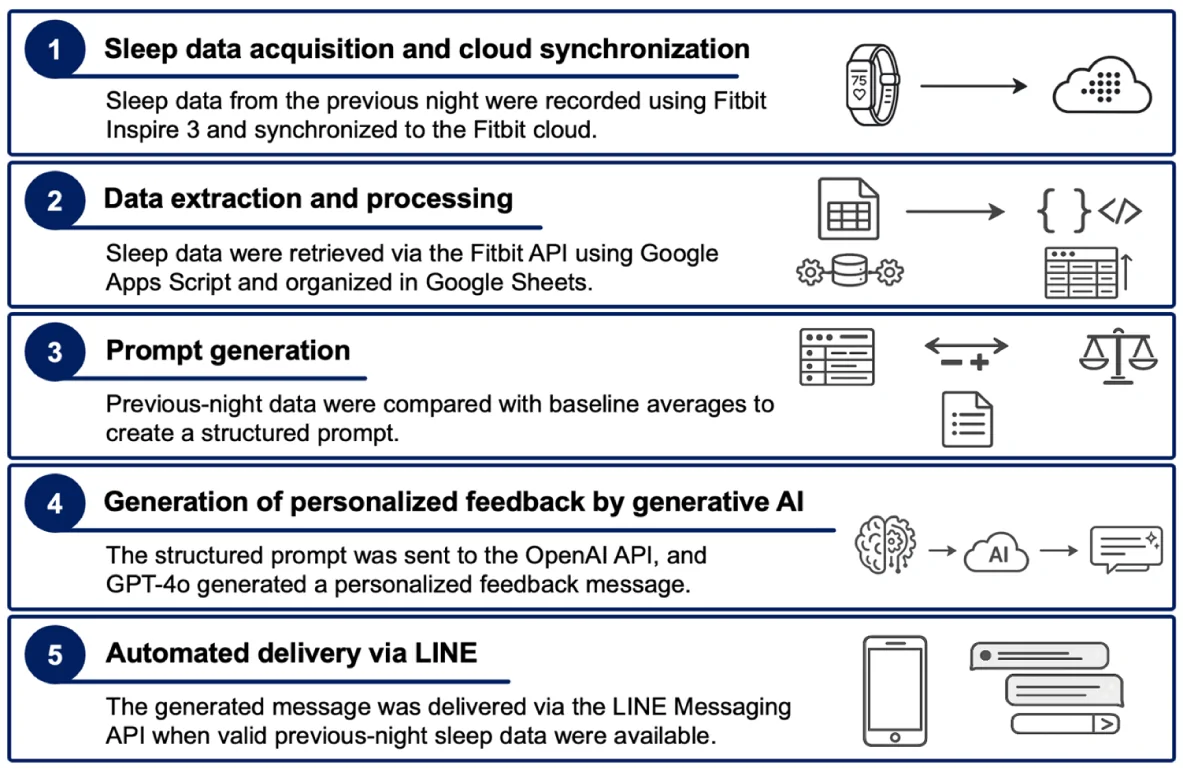
Workflow of the generative AI–based personalized sleep feedback system. Sleep data collected using the Fitbit Inspire 3 were synchronized to the Fitbit cloud, retrieved, processed using Google Apps Script, and organized in Google Sheets. The system compared sleep data from the previous night with baseline averages and converted the results into structured prompts. These prompts were sent to GPT-4o through the OpenAI API to generate personalized feedback messages. The system delivered the generated messages through the LINE Messaging API when valid sleep data from the previous night were available.

Feedback generation and delivery were implemented using Google Apps Script (Google LLC). The system retrieved each participant’s Fitbit sleep data via a user management sheet containing the participant ID, LINE user ID, nickname, group allocation, Fitbit authentication information, and the name of each participant-specific data sheet. Fitbit sleep records were retrieved for each participant and date using the Fitbit Web API sleep end point (/1.2/user/-/sleep/date/{date}.json) with OAuth 2.0 bearer-token authentication. If an access token had expired, the system refreshed the token using the stored refresh token and retried the request. Dates were processed using the Google Apps Script project time zone, which was set to Japan Standard Time during the study. Among the retrieved sleep records, only records flagged as the main sleep episode (isMainSleep = true) were retained. The extracted variables included dateOfSleep, startTime, endTime, timeInBed, minutesAsleep, minutesAwake, efficiency, and sleep-stage summaries for deep, light, REM, and wake stages. These variables were stored in Google Sheets and used for feedback generation and outcome calculation, where applicable.

The input variables used for personalized feedback generation included study nickname (nonidentifying), mean baseline TST, mean baseline sleep efficiency, previous-night bedtime, wake time, TST, deviations from mean baseline TST and sleep efficiency, and previous-night deep sleep and REM sleep durations. These data were transmitted to GPT-4o via the OpenAI API together with a prespecified structured prompt. Information sent to the OpenAI API was restricted to the study nickname and required sleep metrics and did not include direct identifiers such as name, school name, telephone number, email address, LINE display name, LINE user ID, Fitbit authentication information, or parent or guardian information. For message delivery via the LINE Messaging API, LINE user IDs were used to specify recipients; however, these identifiers were managed separately in a correspondence table linking study IDs and were not transmitted to the OpenAI API. The OpenAI Responses API was used. The model, output constraints, prompt structure, data processing workflow, and delivery logic were predefined before trial initiation and were not modified during the study period.

Each generated message consisted of 2 sections: “last night’s sleep data” and “feedback comment.” The “last night’s sleep data” section presented bedtime, wake time, TST, deep sleep duration, and sleep efficiency. The “feedback comment” section included 4 components: a summary of the previous night’s sleep, an explanation of deviation from baseline averages, 1 feasible behavioral suggestion for the day or before the next bedtime, and an encouraging statement. The system prompt was designed to avoid diagnostic statements, treatment instructions, medical judgments, return-to-play decisions, sleep medication advice, individualized recommendations to seek medical care, alarmist language, and blame-oriented phrasing.

When previous-night sleep data were available, feedback was automatically generated and delivered without daily researcher review, following the predefined prompt structure and delivery logic. When valid sleep data were unavailable, no feedback was generated, and no message was delivered; this event was recorded in system logs. Duplicate message delivery on the same day was prevented using delivery-completion flags indexed by date and participant ID. In cases of API rate limiting or server-side errors, retry processing with exponential backoff was implemented. If the response text could not be extracted, the event was logged as an error, and no message was sent.

Prior to trial initiation, the system was tested using dummy data and test users. These tests verified Fitbit data retrieval, token refresh, prompt generation, OpenAI API response, LINE delivery, logging, duplicate prevention, prevention of empty messages, and absence of inappropriate outputs. The input variables, prompt structure, output format, model specification, API configuration, prohibited content rules, failure-handling procedures, and representative outputs are provided in Multimedia Appendix 2. A representative example of feedback delivered to the intervention group is shown in the left panel of Figure 3. The intervention system was a fixed research prototype, and no modifications were made to the prompt structure, data processing workflow, delivery logic, API settings, or delivery configuration during the study period.

**Figure 3:**
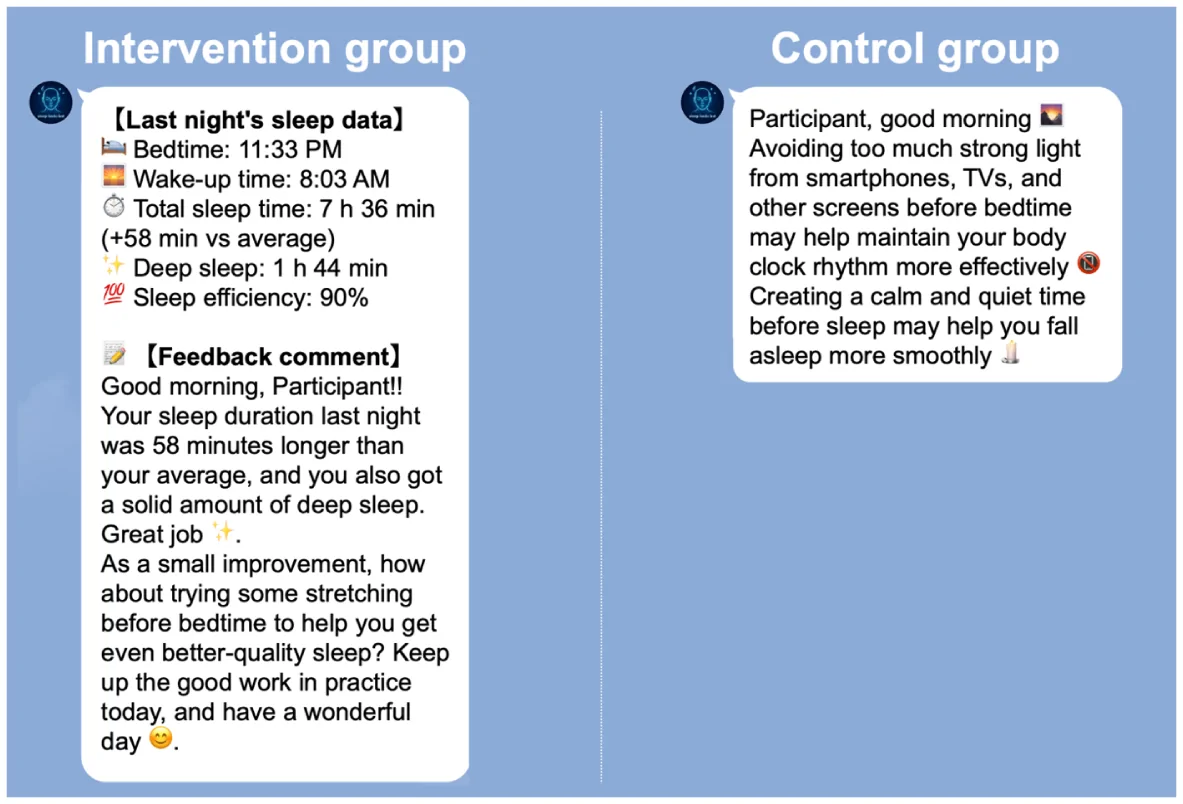
Representative examples of LINE-delivered messages for the intervention and control groups. The left panel presents an example of a personalized sleep feedback message generated by generative AI based on the participant’s objective sleep metrics from the previous night and baseline average values. The right panel presents an example of the general, nonpersonalized sleep information delivered to the control group. For publication purposes, the messages are presented in English translation.

#### Control Group

The control group functioned as an active comparison condition using the same LINE delivery channel and general sleep-related messaging, but without incorporation of prior-night individual sleep data, baseline comparisons, or personalized behavioral recommendations. Participants in the control group received general, nonpersonalized sleep information via LINE each day.

Control-group messages were generated using GPT-4o prior to trial initiation. The research team reviewed all messages for scientific accuracy, ethical acceptability, nonpersonalization, absence of excessive behavioral demands, avoidance of anxiety-inducing language, and exclusion of diagnostic or treatment instructions. Following approval, the messages were stored in Google Sheets as fixed, date-specific content and remained unchanged throughout the study period. The complete set of control-group messages is provided in Multimedia Appendix 2. These messages were fixed, date-specific general sleep information messages, and only the participant’s nickname was inserted at delivery; no Fitbit-derived data, baseline values, subjective condition data, training status, or prior responses were used to generate, select, or modify the messages during the trial.

At the time of delivery, the system retrieved the preassigned message for the corresponding date, inserted only the participant’s nickname, and transmitted the message via LINE. Message content was not modified based on Fitbit data, subjective reports, sleep or training status, or prior responses. Control-group messages did not include prior-night objective sleep data, real-time data interpretation, comparisons with baseline averages, or personalized behavioral recommendations. Aside from nickname insertion, messages were treated as nonpersonalized information in this study. If no message was available for a given date, no message was sent, and the absence was recorded in the study log.

Both groups received sleep-related messages via LINE, but the intervention condition differed from the control condition in several respects. The intervention group received automatically generated personalized feedback based on prior-night Fitbit data and individual baseline averages, whereas the control group received preapproved fixed general sleep information without prior-night data interpretation or individualized behavioral recommendations. Thus, the study compared a multicomponent AI-supported personalized feedback condition with a nonpersonalized messaging condition and did not isolate the effect of GPT-4o, generative AI, or any single intervention component.

### Statistical Analysis

Statistical analyses were conducted using IBM SPSS Statistics (version 30; IBM Corp). Normality was assessed for all continuous variables using the Shapiro-Wilk test. Given the small sample size and nonnormal distribution of several variables, all analyses used nonparametric methods. Descriptive statistics are presented as mean (SD) for age and athletic experience and as median (IQR) for all other variables.

For objective sleep outcomes, the mean of all valid wear nights was calculated for each participant during the baseline period (weeks 1-4) and the intervention period (weeks 6-9), and these values were used as representative estimates for each period. Missing data were not imputed; analyses were therefore based solely on available valid wear nights.

The between-group comparison of change in sleep efficiency constituted the primary preliminary efficacy analysis. Change scores (Δ) were calculated for sleep efficiency and all secondary and exploratory outcomes by subtracting baseline values from postintervention values. Between-group differences in change scores were assessed using the Mann-Whitney *U* test. Hodges-Lehmann (HL) estimates and 95% CIs were calculated to quantify the magnitude and uncertainty of the between-group differences. Between-group differences were defined as the change in the intervention group minus the change in the control group.

Within-group pre-post comparisons were conducted using the Wilcoxon signed rank test. These analyses were considered supplementary and were used to describe the direction of change rather than to infer intervention effects.

Because this was an exploratory pilot trial, no adjustment for multiple comparisons was applied to secondary or exploratory outcomes or to the supplementary within-group comparisons. Accordingly, *P* values for these analyses were considered nominal and exploratory rather than confirmatory. Exact *P* values are reported, except when *P*<.001, in which case inequality notation is used. Interpretation emphasized between-group effect estimates and 95% CIs rather than dichotomous statistical significance. Effect size *r* was calculated as *r*=Z/√N. Effect sizes were interpreted using absolute values, with thresholds of 0.1 to <0.3 considered small, 0.3 to <0.5 considered medium, and ≥0.5 considered large [27].

### Ethical Considerations

This study was approved by the Research Ethics Committee of Nippon Sport Science University (approval number 025-H148) and conducted in accordance with the Declaration of Helsinki. All participants received written information describing the study purpose and procedures, and assent was obtained directly from the players. Because participants were minors, parents or guardians and coaching staff also received written and verbal explanations, and written informed consent was obtained from a parent or guardian for each participant.

To ensure confidentiality, Fitbit data, questionnaire responses, and LINE delivery logs were managed using study identification codes and stored separately from direct identifiers. All research data were stored in password-protected spreadsheets, and access was restricted to the principal investigator and authorized study personnel. The linkage file connecting study IDs with names, LINE user IDs, and other identifiers was stored separately from the analytic dataset.

In this study, Fitbit-derived sleep data were synchronized with the Fitbit cloud, retrieved, and processed using Google Apps Script, and stored in Google Sheets. For participants in the intervention group, the study nickname, prior-night sleep metrics, individual baseline averages, and deviations from those baselines were transmitted to the OpenAI API. Generated feedback messages were delivered to participants via the LINE Messaging API. Data transmitted to the OpenAI API did not include direct identifiers such as name, school name, telephone number, email address, LINE display name, LINE user ID, Fitbit authentication credentials, or parent or guardian information. Although LINE user IDs were required for message delivery, they were managed separately in a linkage file and were not transmitted to the OpenAI API.

The data flow across Fitbit, Google Apps Script, Google Sheets, the OpenAI API, and the LINE Messaging API, as well as associated privacy risks, data protection procedures, the voluntary nature of participation, and withdrawal procedures, were explained in writing to participants and their parents or guardians. Participants received monetary compensation for study participation, funded by the 2025 Nippon Sport Science University Academic Research Grant.

Study briefing, consent procedures, and system setup began on August 18, 2025. The trial was retrospectively registered with the UMIN Clinical Trials Registry on April 7, 2026 (UMIN000061189).

## Results

### Participant Characteristics and Baseline Comparisons

After randomization, 12 participants were allocated to the intervention group and 13 to the control group (Figure 4). No participants withdrew during the study period, and all participants were included in the final analysis. Baseline participant characteristics and outcome measures are summarized in Table 1. No marked baseline imbalance was apparent from the descriptive distributions.

**Figure 4:**
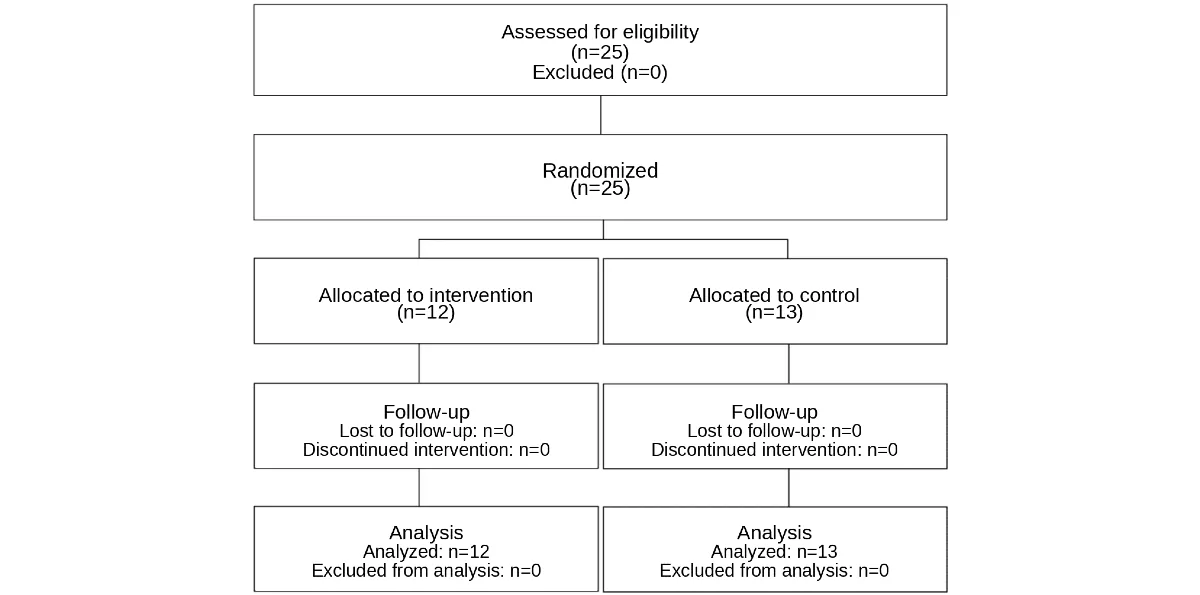
Participant flow diagram for the pilot randomized controlled trial based on the CONSORT (Consolidated Standards of Reporting Trials) statement. The diagram presents the number of participants assessed for eligibility, randomized, allocated to the intervention or control group, followed up, and included in the analysis.

**Table 1:** Baseline characteristics and outcome measures of the intervention and control groups. Data are presented as mean (SD) for age and athletic experience and as median (IQR) for all other variables. Between-group comparisons were performed using the Mann-Whitney U test.

Outcome measure			Intervention group (n=12)		Control group (n=13)		*P* value
Age (years), mean (SD)			16.85 (0.69)		17.00 (0.67)		.82
Athletic experience (years), mean (SD)			9.08 (2.29)		10.10 (1.97)		.23
Objective sleep parameters, median (IQR)							
	Sleep efficiency (%)	87.75 (85.13-88.75)		88.50 (86.50-89.50)		.27	
	Total sleep time (minutes)	347.00 (320.63-381.25)		333.50 (285.00-378.13)		.15	
	Wake after sleep onset (minutes)	49.50 (41.00-64.50)		45.50 (35.75-51.75)		.17	
	Deep sleep duration (minutes)	70.25 (66.88-78.75)		72.50 (65.00-85.63)		.22	
	Light sleep duration (minutes)	197.25 (178.75-219.88)		196.50 (157.13-218.13)		.27	
	REM^a^ sleep duration (minutes)	77.25 (68.00-84.50)		60.25 (49.50-73.88)		.08	
Subjective sleep quality, median (IQR)							
	PSQI-J^b^ total score (points)	7.00 (4.25-8.75)		7.50 (5.00-10.25)		.29	
Sleep behavior, median (IQR)							
	ASSQ-J^c^ SDS^d^ (points)	7.00 (6.00-8.00)		9.00 (6.75-9.25)		.76	
Daytime sleepiness, median (IQR)							
	JESS^e^ total score (points)	10.00 (5.00-16.00)		13.00 (9.75-15.75)		.23	
Mood state, median (IQR)							
	POMS 2^f^ total mood disturbance score (points)	44.50 (30.00-58.50)		54.50 (37.50-64.50)		.46	
Sports injury severity, median (IQR)							
	OSTRC-H-J^g^ severity score (points)	8.00 (0.00-31.00)		3.00 (0.00-22.00)		.54	

^a^REM: rapid eye movement.

^b^PSQI-J: Japanese version of the Pittsburgh Sleep Quality Index.

^c^ASSQ-J: Japanese version of the Athlete Sleep Screening Questionnaire.

^d^SDS: sleep difficulty score.

^e^JESS: Japanese version of the Epworth Sleepiness Scale.

^f^POMS 2: Profile of Mood States, Second Edition.

^g^OSTRC-H-J: Japanese version of the Oslo Sports Trauma Research Center Questionnaire on Health Problems.

### Technical and Operational Feasibility

During the intervention period, participants in the intervention group were scheduled to receive personalized feedback on days when valid prior-night Fitbit sleep data were available. Participants in the control group were scheduled to receive general sleep information at 8 AM each morning. All messages sent via the LINE Messaging API were successfully transmitted in both groups, resulting in a delivery success rate of 100%.

The median number of valid Fitbit wear nights was 26 (IQR 24-28) in the intervention group and 22 (IQR 15-26) in the control group. Questionnaire completion was 100% at both baseline and postintervention assessments. No major technical problems affecting intervention delivery or study continuation were observed. No adverse events related to the intervention or device use occurred, and no privacy breaches, including personal data leakage, were reported.

### Objective Sleep Metrics

Table 2 presents between-group comparisons of change scores for the primary preliminary efficacy outcome and the secondary and exploratory outcomes. Supplementary within-group pre-post comparisons are provided in Table 3 to describe within-group change patterns. Sleep efficiency was the primary preliminary efficacy outcome. TST, WASO, PSQI-J total score, ASSQ-J SDS, and JESS total score were secondary outcomes; deep sleep duration, light sleep duration, REM sleep duration, TMD, and sports injury severity were exploratory outcomes.

A between-group difference was observed for sleep efficiency, the primary preliminary efficacy outcome. The HL estimate of the between-group difference in change was 2.00% (95% CI 0.50%-4.00%), with the change favoring the intervention group (*P*=.046; *r*=0.35). Among the exploratory sleep-stage outcomes, a nominal between-group difference was observed for REM sleep duration (HL estimate 5.25, 95% CI 4.00-11.50 minutes; *P*=.006; *r*=0.51). No between-group differences were detected for changes in TST (HL estimate 21.00, 95% CI −5.50 to 41.50 minutes; *P*=.13), WASO (HL estimate −7.50, 95% CI −16.00 to 2.50 minutes; *P*=.17), deep sleep duration (HL estimate −1.00, 95% CI −8.00 to 6.00 minutes; *P*=.12), or light sleep duration (HL estimate 10.50, 95% CI −7.00 to 28.50 minutes; *P*=.16). Each CI included zero but was wide, and these estimates are imprecise given the small sample.

In the supplementary within-group analyses, changes were observed in several objective sleep measures in the intervention group. Median TST increased from 347.00 minutes (IQR 320.63-381.25) to 363.25 minutes (IQR 316.88-396.00; *P*=.02; *r*=0.53), and WASO decreased from 49.50 minutes (IQR 41.00-64.50) to 37.75 minutes (IQR 33.50-56.00; *P*=.003; *r*=0.67). Sleep efficiency (*P*<.001, *r*=0.78), deep sleep duration (*P*=.001, *r*=0.73), and REM sleep duration (*P*<.001, *r*=0.59) also increased from baseline to postintervention. The corresponding control-group within-group comparisons are reported in Table 3.

**Table 2:** Between-group comparisons of changes in outcome measures from baseline to postintervention. Change (Δ) was calculated as the postintervention value minus the baseline value. Between-group differences were calculated as the change in the intervention group minus the change in the control group. HL^a^ estimates and 95% CIs are shown for between-group differences in change scores. Between-group comparisons were performed using the Mann-Whitney U test. No adjustment for multiple comparisons was performed. *P* values for secondary and exploratory outcomes are therefore nominal and should not be interpreted as confirmatory statistical significance. Interpretation should emphasize the HL estimates and 95% CIs.

Outcome measure	Intervention Δ, median (IQR)	Control Δ, median (IQR)	Between-group difference, HL estimate (95% CI)	*P* value	*r*
Sleep efficiency (%)	1.75 (0.63 to 3.00)	0.00 (−1.63 to 0.75)	2.00 (0.50 to 4.00)	.046	0.35
Total sleep time (minutes)	15.50 (−4.75 to 29.25)	−8.50 (−17.25 to 13.38)	21.00 (−5.50 to 41.50)	.13	0.24
Wake after sleep onset (minutes)	−4.00 (−11.38 to −0.63)	−0.25 (−5.88 to 7.63)	−7.50 (−16.00 to 2.50)	.17	0.21
Deep sleep duration (minutes)	4.50 (1.13 to 7.75)	6.25 (0.13 to 9.50)	−1.00 (−8.00 to 6.00)	.12	0.25
Light sleep duration (minutes)	0.25 (−3.38 to 13.88)	−3.00 (−25.63 to 6.25)	10.50 (−7.00 to 28.50)	.16	0.21
REM^b^ sleep duration (minutes)	7.25 (1.13 to 11.38)	−1.50 (−6.00 to 0.75)	5.25 (4.00 to 11.50)	.006	0.51
PSQI-J^c^ total score (points)	−2.00 (−2.75 to −1.25)	−0.50 (−3.25 to 1.75)	−2.00 (−4.00 to −1.00)	.04	−0.41
ASSQ-J^d^ SDS^e^ (points)	−1.50 (−2.00 to 0.00)	0.50 (−1.75 to 1.75)	−2.00 (−4.00 to −0.10)	.046	−0.34
JESS^f^ total score (points)	−2.00 (−4.00 to 0.00)	−2.00 (−3.75 to 2.25)	−1.00 (−5.00 to 2.00)	.22	−0.25
POMS 2^g^ TMD^h^ score (points)	−11.50 (−18.75 to −4.75)	0.00 (−10.75 to 13.25)	−13.50 (−26.00 to −1.00)	.01	−0.52
OSTRC-H-J^i^ severity score (points)	0.00 (−8.00 to 0.00)	0.00 (−6.00 to 20.50)	−6.00 (−28.00 to 0.00)	.20	−0.27

^a^HL: Hodges-Lehmann.

^b^REM: rapid eye movement.

^c^PSQI-J: Japanese version of the Pittsburgh Sleep Quality Index.

^d^ASSQ-J: Japanese version of the Athlete Sleep Screening Questionnaire.

^e^SDS: sleep difficulty score.

^f^JESS: Japanese version of the Epworth Sleepiness Scale.

^g^POMS 2: Profile of Mood States, Second Edition.

^h^TMD: total mood disturbance.

^i^OSTRC-H-J: Japanese version of the Oslo Sports Trauma Research Center Questionnaire on Health Problems.

**Table 3:** Supplementary within-group comparisons from baseline to postintervention. Within-group comparisons between baseline and postintervention values were performed using the Wilcoxon signed rank test. These supplementary analyses examined the direction of change within each group and did not serve as the primary basis for evaluating intervention effects. Because no adjustment was made for multiple supplementary comparisons, the corresponding *P* values are nominal and exploratory.

Outcome measure and group		Baseline, median (IQR)	Postintervention, median (IQR)	*P* value	Effect size, *r*
Sleep efficiency (%)					
	Intervention	87.75 (85.13-88.75)	89.75 (86.75-91.00)	<.001	0.78
	Control	88.50 (86.50-89.50)	87.50 (86.25-89.00)	.26	0.22
Total sleep time (minutes)					
	Intervention	347.00 (320.63-381.25)	363.25 (316.88-396.00)	.02	0.53
	Control	333.50 (285.00-378.13)	328.00 (298.50-387.38)	.29	0.19
Wake after sleep onset (minutes)					
	Intervention	49.50 (41.00-64.50)	37.75 (33.50-56.00)	.003	0.67
	Control	45.50 (35.75-51.75)	46.25 (36.25-55.25)	.20	0.28
Deep sleep duration (minutes)					
	Intervention	70.25 (66.88-78.75)	76.25 (72.75-79.50)	.001	0.73
	Control	72.50 (65.00-85.63)	83.00 (69.63-96.25)	.10	0.41
Light sleep duration (minutes)					
	Intervention	197.25 (178.75-219.88)	202.50 (179.88-224.13)	.15	0.28
	Control	196.50 (157.13-218.13)	183.00 (157.00-226.00)	.49	0.01
REM^a^ sleep duration (minutes)					
	Intervention	77.25 (68.00-84.50)	80.75 (75.13-93.63)	<.001	0.59
	Control	60.25 (49.50-73.88)	63.50 (52.25-74.25)	.18	0.31
PSQI-J^b^ total score (points)					
	Intervention	7.00 (4.25-8.75)	4.00 (2.25-6.00)	.002	−0.86
	Control	7.50 (5.00-10.25)	7.00 (4.75-9.50)	.28	−0.31
ASSQ-J^c^ SDS^d^ (points)					
	Intervention	7.00 (6.00-8.00)	5.00 (4.00-6.00)	.002	−0.84
	Control	9.00 (6.75-9.25)	8.00 (6.00-9.50)	.75	−0.09
JESS^e^ total score (points)					
	Intervention	10.00 (5.00-16.00)	8.00 (7.00-13.00)	.13	−0.42
	Control	13.00 (9.75-15.75)	11.00 (8.75-16.75)	.51	−0.19
POMS 2^f^ total mood disturbance score (points)					
	Intervention	44.50 (30.00-58.50)	35.50 (7.00-45.50)	.01	−0.71
	Control	54.50 (37.50-64.50)	51.50 (43.00-54.75)	.65	−0.13
OSTRC-H-J^g^ severity score (points)					
	Intervention	8.00 (0.00-31.00)	0.00 (0.00-17.00)	.09	−0.47
	Control	3.00 (0.00-22.00)	0.00 (0.00-32.00)	.48	−0.20

^a^REM: rapid eye movement.

^b^PSQI-J: Japanese version of the Pittsburgh Sleep Quality Index.

^c^ASSQ-J: Japanese version of the Athlete Sleep Screening Questionnaire.

^d^SDS: sleep difficulty score.

^e^JESS: Japanese version of the Epworth Sleepiness Scale.

^f^POMS 2: Profile of Mood States, Second Edition.

^g^OSTRC-H-J: Japanese version of the Oslo Sports Trauma Research Center Questionnaire on Health Problems.

### Subjective Sleep Quality

The change in PSQI-J total score was −2.00 (IQR −2.75 to −1.25) in the intervention group and −0.50 (IQR −3.25 to 1.75) in the control group. A nominal between-group difference was observed (HL estimate −2.00, 95% CI −4.00 to −1.00; *P*=.04; *r*=−0.41). In the supplementary within-group analysis, the PSQI-J total score decreased from baseline to postintervention in the intervention group (*P*=.002; *r*=−0.86), whereas no comparable change was detected in the control group.

### Sleep Behavior and Daytime Sleepiness

The change in SDS was −1.50 (IQR −2.00 to 0.00) in the intervention group and 0.50 (IQR −1.75 to 1.75) in the control group. A nominal between-group difference was observed (HL estimate −2.00, 95% CI −4.00 to −0.10; *P*=.046; *r*=−0.34). In the supplementary within-group analysis, the SDS decreased from baseline to postintervention in the intervention group (*P*=.002; *r*=−0.84), whereas no corresponding pre-post change was observed in the control group.

For daytime sleepiness measured using the JESS, no between-group difference was observed in change scores (*P*=.22). The within-group comparisons yielded *P*=.13 in the intervention group and *P*=.51 in the control group.

### Mood State

The change in TMD score was −11.50 (IQR −18.75 to −4.75) in the intervention group and 0.00 (IQR −10.75 to 13.25) in the control group. A nominal between-group difference with a large effect size was observed (HL estimate −13.50, 95% CI −26.00 to −1.00; *P*=.01; *r*=−0.52). In the supplementary within-group analysis, the TMD score decreased from baseline to postintervention in the intervention group (*P*=.01; *r*=−0.71), whereas no corresponding pre-post change was observed in the control group.

### Sports Injury Severity

For sports injury severity assessed using the modified OSTRC-H-J, no between-group difference was observed in change scores (HL estimate −6.00, 95% CI −28.00 to 0.00; *P*=.20; *r*=−0.27). The within-group comparisons yielded *P*=.09 (*r*=−0.47) in the intervention group and *P*=.48 (*r*=−0.20) in the control group.

## Discussion

### Principal Results

This pilot randomized controlled trial evaluated the technical and operational feasibility of a multicomponent AI-supported mHealth intervention that integrated prior-night wearable-derived sleep data, individual baseline comparisons, automated personalized feedback generated using generative AI, behavioral recommendations, and LINE-based delivery among high school female soccer players. The study also examined preliminary changes in sleep-related outcomes, with sleep efficiency designated as the primary preliminary efficacy outcome. Other sleep-related measures were evaluated as secondary or exploratory outcomes, while mood state and sports injury severity were exploratory outcomes.

Regarding technical and operational feasibility, the message delivery success rate was 100% in both groups, and the questionnaire completion rate was also 100%. No major technical problems, adverse events, or privacy breaches occurred. These findings support the technical and operational feasibility of the system in this single-team setting.

For sleep efficiency, the primary preliminary efficacy outcome, the between-group change favored the intervention group (HL estimate 2.00%, 95% CI 0.50–4.00). This estimate should be interpreted cautiously given the small sample, the borderline *P* value (*P*=.046), and the retrospective trial registration. Nominal between-group differences were also observed in the PSQI-J and SDS, both secondary outcomes, and in TMD, an exploratory outcome. In contrast, no between-group differences were observed for TST, WASO, deep sleep duration, light sleep duration, daytime sleepiness, or sports injury severity. Because no adjustment was made for multiple comparisons, *P* values for the secondary and exploratory outcomes are nominal and should not be interpreted as confirmatory statistical significance. Given the exploratory pilot design, these findings represent preliminary intervention-associated differences rather than evidence of established effectiveness and are best treated as hypothesis-generating signals that may help identify outcomes worth evaluating in a future definitive trial.

### Comparison With Prior Work

Regarding sleep-related outcomes, the between-group change in sleep efficiency, the primary preliminary efficacy outcome, favored the intervention group. Nominal between-group differences were also observed in the PSQI-J and SDS, both secondary outcomes. The direction of these preliminary findings is consistent with the sleep-related changes reported by Barley and Scullin [10] following a behavioral sleep intervention. A nominal between-group difference was also observed in REM sleep duration. This finding should be regarded as purely exploratory because REM sleep duration was estimated using a consumer wearable device rather than polysomnography. This pattern differs from the wearable-based intervention in young adults reported by Fucito et al [28]; however, direct comparison is limited by differences in populations, intervention content, and outcome measures.

Previous sleep interventions have often relied on standardized education, sleep hygiene instruction, or self-monitoring, whereas the present intervention translated each participant’s prior-night wearable-derived sleep data into individualized feedback delivered the following morning. However, the present study did not assess the mechanisms underlying the observed between-group differences. In particular, attention to sleep, reflection on sleep behavior, adherence to the behavioral suggestions, and subsequent behavioral changes were not measured. Because the intervention components were delivered as a bundle, the present design cannot determine whether the observed differences were related to prior-night wearable-data integration, comparison with individual baseline values, personalization, message timing, behavioral specificity, supportive framing, automated message generation, or a combination of these components.

No between-group difference was observed in TST. This finding may reflect structural constraints among high school students, including daily training demands and commuting time [3], which limit opportunities to extend sleep duration.

Regarding sleep behavior and daytime sleepiness, a nominal between-group difference was observed in SDS. This finding is consistent with prior reports that sleep-focused interventions can produce changes in sleep-related behaviors in athletes [8]. The intervention provided personalized behavioral suggestions, such as optimizing the sleep environment and limiting smartphone use before bedtime. However, whether participants read, understood, or acted on these suggestions was not assessed. No between-group difference was observed in JESS. Daytime sleepiness is related to sleep restriction in adolescents [29] and to insufficient sleep more broadly [30]. The absence of a between-group difference in TST may therefore provide one possible context for the lack of a corresponding difference in daytime sleepiness.

Regarding mood state, a nominal between-group difference was observed in TMD, an exploratory outcome. Sleep quality and mood state have been reported to be related in athletes [31], providing context for the concurrent between-group differences observed in selected sleep-related outcomes and TMD in this study. However, the present study did not determine whether the sleep-related differences were causally related to the TMD difference or whether specific intervention components, such as positive feedback or numerical presentation of sleep status, contributed to this finding. The mechanism underlying the TMD difference therefore remains uncertain.

Regarding sports injury severity, no between-group difference was observed in changes in the modified OSTRC-H-J score. This may reflect the multifactorial nature of sports injuries, for which short-term sleep-focused interventions alone may be insufficient to produce detectable changes [32]. Injury occurrence and exacerbation are influenced by cumulative and long-term factors, including accumulated fatigue and delayed recovery processes. Therefore, a 1-month intervention period may have been too short for measurable changes in injury-related burden to emerge. Although no between-group difference was observed in sports injury severity, between-group differences were observed in selected sleep-related outcomes and mood state. Whether these differences translate into long-term reductions in injury burden remains uncertain and should be examined in longer-duration trials with adequate statistical power.

The system automated Fitbit data processing, generation of individualized feedback, and LINE-based message delivery throughout the intervention period. This workflow reduced the need for manual daily data processing and message preparation by the research team. However, participant engagement, satisfaction, perceived burden, acceptability, and willingness to continue using the system were not assessed. Therefore, the present findings do not establish broader user acceptability or sustained use.

### Limitations

This study has several limitations. First, participants were drawn from a single high school female soccer team, and the sample size was small. Accordingly, the intervention should be tested in populations that vary by age, sport, and sex to enhance generalizability. In addition, because individual randomization occurred within the same team, there is a risk of contamination. Personalized feedback provided to the intervention group, as well as behavioral sleep recommendations, may have been shared with the control group through daily conversations or screen sharing. As the extent of message sharing or intervention contamination was not assessed, the degree of cross-arm contamination could not be quantified. In addition, because all participants were recruited from a single team, individual responses may not have been fully independent. Team-level factors such as training schedules, coaching environment, shared routines, and peer communication may have influenced sleep behavior and intervention responses. Owing to the small sample size and single-team design, clustering effects were not modeled statistically. These team-level social dynamics and potential cross-arm contamination may have attenuated the observed between-group differences.

Second, several potential confounding factors were not adequately controlled. Daily training load, academic stress, and dietary intake were not monitored and may have influenced changes in sleep and mood outcomes. In addition, the intervention was multicomponent: it combined prior-night Fitbit data, comparisons with individual baseline values, personalized numerical feedback, behavioral recommendations, encouraging language, automated GPT-4o generation, and LINE-based delivery following data synchronization. By contrast, the control group received fixed, prereviewed, nonpersonalized messages at 8 AM and did not incorporate prior-night individual data or personalized behavioral recommendations. Control-group messages were also generated using GPT-4o before trial initiation. Therefore, this study did not compare generative AI with a non-AI condition, and the observed between-group differences cannot be attributed specifically to GPT-4o, generative AI, or any single intervention component.

Third, this study was designed as an exploratory pilot trial. Its primary purpose was to assess technical and operational feasibility and estimate the direction and magnitude of preliminary effects in preparation for a full-scale trial. A formal sample size calculation based on power analysis for confirmatory hypothesis testing was not performed. Accordingly, *P* values should not be interpreted as confirmatory evidence of efficacy. For secondary and exploratory outcomes, as well as supplementary within-group comparisons, *P* values are nominal because no adjustment for multiple comparisons was applied. The observed between-group differences and effect-size estimates should therefore be regarded as hypothesis-generating. They may help identify candidate outcomes and inform effect-size assumptions and sample-size planning for a future definitive trial but should not be treated as precise estimates of treatment effects.

Fourth, study procedures began on August 18, 2025, and the trial was retrospectively registered in the UMIN Clinical Trials Registry on April 7, 2026 (UMIN000061189). As a result, the findings should be interpreted as those of an exploratory pilot study rather than confirmatory evidence derived from a prospectively registered protocol. Future full-scale effectiveness trials should be conducted as protocol-driven randomized controlled trials with prospective registration prior to initiation.

Fifth, sleep-stage data derived from Fitbit represent algorithm-based estimates from a consumer wearable device and do not have the same level of accuracy as polysomnography. Therefore, findings related to deep sleep duration and REM sleep duration should be interpreted with caution and regarded as purely exploratory. Taken together, the small sample, exploratory design, retrospective registration, and multiple statistical comparisons limit causal interpretation of the observed between-group differences.

Sixth, participant engagement and acceptability were not assessed. Message opening or reading, viewing duration, comprehension, satisfaction, perceived burden, and willingness to continue using the system were not measured. Therefore, the feasibility findings are limited to technical and operational dimensions and do not establish participant acceptability or sustained use.

Future definitive trials should prospectively evaluate the outcomes that showed preliminary between-group differences in this pilot study, while also examining longer-term sports injury severity, injury-related burden, and athletic performance. In multiteam studies, cluster randomization at the team level should be considered to reduce cross-arm contamination arising from peer communication and message sharing. Larger samples will be required to provide sufficiently precise estimates and to evaluate effectiveness. On this basis, further development and evaluation of the multicomponent AI-supported mHealth intervention examined here should be considered, including its effects on psychological and physical outcomes and injury-related burden.

### Conclusion

This exploratory pilot randomized controlled trial demonstrated the technical and operational feasibility of a multicomponent AI-supported mHealth intervention in a single high school female soccer team. Favorable, hypothesis-generating between-group differences were observed in sleep efficiency (the primary preliminary efficacy outcome) and in selected secondary and exploratory sleep and mood outcomes, whereas no between-group differences were observed in TST, daytime sleepiness, or sports injury severity. These findings should not be interpreted as evidence of established effectiveness. Interpretation is limited by the small sample, single-team setting, possible within-team contamination, retrospective trial registration, and the absence of adjustment for multiple comparisons. Fitbit-derived sleep-stage findings, including REM and deep sleep duration, should be regarded as exploratory because these algorithm-derived estimates do not have the diagnostic precision of polysomnography. Confirmation requires a prospectively registered, adequately powered, longer-term randomized trial with improved control of contamination and direct assessment of participant engagement and acceptability.

## Appendix

Multimedia Appendix 1 CONSORT-EHEALTH checklist.

Multimedia Appendix 2 Technical specification, prompt design, quality assurance, and representative outputs of the personalized sleep feedback system.

Multimedia Appendix 3 Use of generative AI during manuscript preparation.

Multimedia Appendix 4 Adaptation and operational use of the Japanese version of the Oslo Sports Trauma Research Center Questionnaire on Health Problems (OSTRC-H-J) in this study.

## Abbreviations

ASSQ-J: Japanese version of the Athlete Sleep Screening Questionnaire
CONSORT: Consolidated Standards of Reporting Trials
CONSORT-EHEALTH: Consolidated Standards of Reporting Trials of Electronic and Mobile Health Applications and Online Telehealth
HL: Hodges-Lehmann
JESS: Japanese version of the Epworth Sleepiness Scale
mHealth: mobile health
OSTRC-H-J: Japanese version of the Oslo Sports Trauma Research Center Questionnaire on Health Problems
POMS 2: Profile of Mood States, Second Edition
PSQI-J: Japanese version of the Pittsburgh Sleep Quality Index
REM: rapid eye movement
SDS: sleep difficulty score
TMD: total mood disturbance
TST: total sleep time
WASO: wake after sleep onset

## Data Availability

The individual-level datasets generated and analyzed during this study are not publicly available because the participants were minors and members of a single athletic team, creating a risk of reidentification and associated ethical restrictions on data sharing. Anonymized aggregate data or a limited subset of data may be made available from the corresponding author on reasonable request, subject to approval by the institutional research ethics committee, authorization from the participating institution, and a justified research purpose.

The full source code of the intervention system, API configuration details, authentication credentials, LINE user identifiers, Fitbit authentication data, and the linkage file connecting study identifiers to participant information are not publicly available due to privacy protection requirements and security considerations associated with third-party service integration. To support reproducibility within these constraints, the primary prompt structure, input variables, overview of the delivery logic, safety controls, quality assurance procedures, representative outputs, and the operational use of the modified Japanese version of the Oslo Sports Trauma Research Center Questionnaire on Health Problems (OSTRC-H-J) are provided in Multimedia Appendices 1-4.
