# Supplementary material for: Feasibility and Preliminary Effects of AI-Generated Personalized Sleep Feedback in High School Female Soccer Players: Pilot Randomized Controlled Trial
Source: JMIR Form Res. 2026 Oct 1;10:e101615. doi: 10.2196/101615 (PMC13673175; doi:10.2196/101615)
Supplement: Supplementary file 2 [file formative_v10i1e101615_app2.docx]

**Multimedia Appendix 2**

**Technical Specification, Prompt Design, Quality Assurance, and Representative Outputs of the Personalized Sleep Feedback System**

**Overview**

This appendix describes the technical architecture, data flow, prompt framework, quality assurance procedures, safety controls, and representative outputs of the personalized sleep feedback system used in this pilot randomized controlled trial. The system collected objective sleep data from a wearable device, generated participant-specific feedback using a large language model, and delivered messages through a mobile messaging platform.

The intervention consisted of personalized sleep feedback delivered to the intervention group on days with valid prior-night Fitbit sleep data, and daily general sleep information delivered to the control group. Although the Google Apps Script project included auxiliary functions for administrative testing, weekly report generation, nightly reminders, and automated responses to LINE messages, these functions were not activated during the trial and did not constitute components of the evaluated intervention.

**System Architecture**

The system comprised four main components: (1) Fitbit Inspire 3 for continuous sleep monitoring, (2) Google Sheets and Google Apps Script for participant management, data storage, processing, and workflow automation, (3) the OpenAI application programming interface (API) with GPT-4o for natural language feedback generation, and (4) the LINE Messaging API for automated message delivery [1-6].

Participant information was maintained in a Google Sheets–based user management table. This table included study-related system fields such as participant identifiers, group allocation, nickname, LINE user identifiers, Fitbit authentication information, and participant-specific sleep data sheet references. Security-sensitive identifiers, authentication tokens, spreadsheet identifiers, and API credentials are not disclosed in this appendix.

**Data Acquisition and Preprocessing**

Objective sleep data were obtained from Fitbit Inspire 3 via the Fitbit API and stored in participant-specific Google Sheets. Only records flagged as the main sleep episode (isMainSleep = true) were retained for feedback generation. Extracted variables included sleep date, bedtime, wake time, total sleep time, wake after sleep onset, time in bed, sleep efficiency, and sleep-stage measures, including deep sleep, light sleep, rapid eye movement sleep, and wake minutes.

During the baseline period, participant-specific mean values were computed and used as reference points for personalized comparisons during the intervention period. These baseline averages provided contextualization for each participant’s prior-night sleep data.

**OpenAI Application Programming Interface and Model Configuration**

Personalized feedback messages were generated using GPT-4o via the OpenAI Responses API. The model specification, prompt template, output format, token limit, data processing workflow, and message delivery logic were fixed prior to the intervention and remained stable throughout the trial.

The maximum output length was set to 800 tokens. Security-sensitive configuration values, including API keys, authentication tokens, spreadsheet identifiers, folder identifiers, and LINE channel credentials, are not disclosed.

**Input Variables for Personalized Feedback**

For participants in the intervention group, the system retrieved prior-night sleep metrics following Fitbit synchronization and combined them with participant-specific baseline averages. The following input variables were used:

- Participant nickname
- Mean baseline sleep duration
- Mean baseline sleep efficiency
- Bedtime on the previous night
- Wake time on the previous night
- Actual sleep duration on the previous night
- Difference in sleep duration from baseline
- Sleep efficiency on the previous night
- Difference in sleep efficiency from baseline
- Deep sleep duration on the previous night
- Rapid eye movement sleep duration on the previous night

Sleep duration and sleep-stage variables were expressed in hours and minutes. Sleep efficiency was expressed as a percentage. Deviations from baseline were reported as signed differences.

**Personalized Feedback Generation**

For the intervention group, structured prompts were automatically generated and transmitted to GPT-4o via the OpenAI API. The model was instructed to assume the role of a sleep and conditioning specialist for high school female soccer players and to generate supportive, positive, and easily understandable morning feedback.

The output was required to include two sections: “last night’s sleep data” and “feedback comment.” The sleep data section reported bedtime, wake time, actual sleep duration, deep sleep duration, and sleep efficiency. The feedback comment included the following components:

- A brief summary of the prior-night sleep condition
- An explanation of deviation from baseline values
- One practical and feasible recommendation for the day or upcoming night
- A brief encouraging closing statement

The feedback comment was constrained to approximately 150 to 200 Japanese characters, required natural inclusion of the participant’s nickname once, and was formatted into two to four visually distinct lines.

**Safety Controls and Prohibited Content**

The prompt and system design explicitly excluded diagnostic and therapeutic content. Generated feedback was instructed not to include:

- Diagnostic statements
- Treatment instructions
- Medical decision-making
- Medication-related advice, including sleep medications
- Return-to-play decisions
- Instructions to seek medical care
- Excessive warnings
- Blame-oriented language
- Language that could increase anxiety

The system further constrained outputs to a single actionable suggestion and required a consistently supportive and encouraging tone.

**Representative Intervention Prompt Framework**

The following is a representative English description of the prompt framework. The original prompt was written in Japanese and automatically populated with participant-specific values.

You are a sleep and conditioning specialist for high school female soccer players.

Please provide a supportive, positive, and easy-to-understand morning feedback message based on the following sleep data.

Participant nickname: {{nickname}}

Baseline data:

- Mean baseline sleep duration: {{baseline_sleep_duration}}
- Mean baseline sleep efficiency: {{baseline_sleep_efficiency}}

Previous-night sleep data:

- Bedtime: {{bedtime}}
- Wake-up time: {{wake_time}}
- Actual sleep duration: {{sleep_duration}} {{difference_from_baseline_sleep_duration}}
- Sleep efficiency: {{sleep_efficiency}} {{difference_from_baseline_sleep_efficiency}}
- Deep sleep duration: {{deep_sleep_duration}}
- REM sleep duration: {{rem_sleep_duration}}

Output format:
<sleep feedback>

[Last night's sleep data]

- Bedtime
- Wake-up time
- Actual sleep duration
- Deep sleep duration
- Sleep efficiency

<Feedback comment>

Write approximately 150–200 Japanese characters. Include the participant’s nickname once in a natural manner. Use 2–4 visually clear lines.

Include:

1. A positive evaluation of last night’s sleep
2. An interpretation based on objective values and baseline averages
3. One feasible suggestion for today or tonight
4. An encouraging closing sentence

Do not include diagnosis, treatment instructions, medication advice, return-to-play decisions, excessive warnings, blame-oriented language, or statements that may increase anxiety.

**Representative Intervention Output**

The following example is an English translation prepared for publication. The actual intervention messages were delivered in Japanese.

<sleep feedback>

[Last night's sleep data]

- 🛌 Bedtime: 11:33 PM
- 🌅 Wake-up time: 8:03 AM
- ⏱️ Actual sleep time: 7 h 36 min (+58 min vs baseline average)
- ✨ Deep sleep: 1 h 44 min
- 💯 Sleep efficiency: 90%

📝 <Feedback comment>
Good morning, {{nickname}}!
Your sleep duration last night was 58 minutes longer than your baseline average, and you also achieved a solid amount of deep sleep. Great job ✨
As a small improvement, you might try some gentle stretching before bedtime to help prepare your body for another good night of sleep.
Keep up the good work in practice today, and have a wonderful day 😊

**Control-Group Message Design**

Participants in the control group received general, nonpersonalized sleep-related information through the same LINE-based delivery channel. These messages were pregenerated using GPT-4o; however, no participant-specific Fitbit data, subjective condition data, sleep status, training status, or prior responses were used to modify content.

Control-group messages were developed according to the following principles:

- Content remained general and nonpersonalized
- Messages could include general sleep hygiene, daily rhythm, environmental adjustment, relaxation strategies, and general wellness information
- No individualized interpretation or advice based on participant data was permitted
- Diagnostic language, treatment instructions, and strong behavior-change directives were avoided
- All messages were reviewed by the researchers prior to delivery

Following review, approved messages were stored in Google Sheets as date-specific fixed entries. During delivery, the system retrieved the message corresponding to the target date and inserted only the participant’s nickname. Aside from this insertion, control-group messages were treated as nonpersonalized content.

**Representative Control Output**

The following example is an English translation prepared for publication. The actual control messages were delivered in Japanese.

Good morning, {{nickname}} 🌄
Avoiding too much strong light from smartphones, TVs, and other screens before bedtime may help maintain your body clock rhythm more effectively 📵
Creating a calm and quiet time before sleep may help you wind down more comfortably 🕯️

**Delivery Workflow**

Each morning during the intervention period, the system verified whether Fitbit data had been synchronized. A time-based Google Apps Script trigger executed this process at fixed intervals beginning in the morning.

For intervention-group participants, the system retrieved Fitbit sleep data for the target date, confirmed the presence of a valid main sleep record in the participant-specific sheet, generated a personalized feedback message using the OpenAI API, delivered the message via the LINE Messaging API, and stored the output in a feedback log. When valid previous-night Fitbit sleep data were unavailable, the system did not generate personalized feedback, and no message was delivered for that cycle.

For control-group participants, the system retrieved the preapproved message for the target date, inserted the participant’s nickname, and delivered it via the same LINE Messaging API. When no corresponding message was available, delivery was skipped, and the event was logged.

Duplicate daily delivery was prevented using date- and participant-specific delivery-status flags.

**Failure Handling and Logging**

The system incorporated multiple failure-handling procedures. OpenAI API calls included retry mechanisms for transient rate-limit and server-side errors. If responses did not contain extractable text, the error was logged, and no message was sent.

Fitbit API errors, missing participant sheets, missing sleep records, token refresh failures, missing control messages, and LINE push delivery failures were also logged. When valid previous-night Fitbit sleep data were unavailable for intervention participants, no personalized feedback was generated for that cycle. All generated intervention messages were stored in a centralized feedback management sheet. Delivery-status flags were recorded using date- and participant-specific script properties to prevent duplication.

**Quality Assurance and Pretrial Testing**

Before the intervention period, the research team conducted test runs using dummy data and test users. These tests verified Fitbit data retrieval, token refresh procedures, prompt construction, OpenAI API response generation, LINE message delivery, feedback logging, duplicate delivery prevention, missing-message handling, and the absence of blank or inappropriate outputs.

Control-group message delivery was also tested to confirm date-based retrieval and correct nickname insertion. Following testing, the prompt structure, model configuration, output format, data processing pipeline, delivery logic, and message review procedures were finalized before study initiation.

**Monitoring and Implementation Logging**

The system recorded key implementation indicators, including message delivery status, Fitbit synchronization attempts, generated feedback outputs, and error logs. In the main study, technical and operational feasibility outcomes were summarized using message delivery success rate, valid Fitbit wear nights, questionnaire completion rate, technical problems, adverse events, and privacy breaches.

**Nonpublic Implementation Details**

The full source code is not publicly available because it contains service configuration parameters, API connection settings, spreadsheet and folder identifiers, authentication information, token management procedures, and third-party integration details that could compromise system security or participant privacy.

To support transparency and reproducibility while maintaining security and confidentiality, this appendix reports the system architecture, data flow, input variables, prompt framework, output format, safety constraints, quality assurance procedures, failure-handling logic, and representative outputs at a level sufficient for methodological interpretation.

**References**

1. Fitbit. Inspire 3 health and fitness tracker. Fitbit. Accessed April 17, 2026. <https://www.fitbit.com/global/us/products/trackers/inspire3>
2. Google for Developers. Apps Script. Google. Accessed April 17, 2026. <https://developers.google.com/apps-script>
3. Google for Developers. Google Apps Script overview. Google. Accessed April 17, 2026. <https://developers.google.com/apps-script/overview>
4. LINE Developers. Messaging API overview. LINE. Accessed April 17, 2026. <https://developers.line.biz/en/docs/messaging-api/overview/>
5. OpenAI. OpenAI platform. OpenAI. Accessed April 17, 2026. <https://platform.openai.com/>
6. OpenAI. Models. OpenAI API reference. Accessed April 17, 2026. <https://platform.openai.com/docs/api-reference/models>
7. Kawasaki Y, Kasai T, Sakurama Y, Sekiguchi A, Kitamura E, Midorikawa I, Shiroshita N, Kawana F, Ogasawara E, Kitade M, Koikawa N, Matsuda T. Evaluation of sleep parameters and sleep staging (slow wave sleep) in athletes by Fitbit Alta HR, a consumer sleep tracking device. Nat Sci Sleep. 2022;14:819-827. doi:10.2147/NSS.S351274. PMID:35502231.
8. Sargent C, Lastella M, Romyn G, Versey NGN, Miller DJ, Roach GD. How well does a commercially available wearable device measure sleep in young athletes? Chronobiol Int. 2018;35(6):754-758. doi:10.1080/07420528.2018.1466800. PMID:29750580.
9. Takeuchi H, Suwa K, Kishi A, Nakamura T, Yoshiuchi K, Yamamoto Y. The effects of objective push-type sleep feedback on habitual sleep behavior and momentary symptoms in daily life: mHealth intervention trial using a health care Internet of Things system. JMIR Mhealth Uhealth. 2022;10(10):e39150. doi:10.2196/39150. PMID:36201383.
10. Jakowski S, Stork M. Effects of sleep self-monitoring via app on subjective sleep markers in student athletes. Somnologie (Berl). 2022;26(4):244-251. doi:10.1007/s11818-022-00395-z. PMID:36311283.
