# Supplementary material for: Feasibility and Preliminary Effects of AI-Generated Personalized Sleep Feedback in High School Female Soccer Players: Pilot Randomized Controlled Trial
Source: JMIR Form Res. 2026 Oct 1;10:e101615. doi: 10.2196/101615 (PMC13673175; doi:10.2196/101615)
Supplement: Supplementary file 3 [file formative_v10i1e101615_app3.docx]

**Multimedia Appendix 3**

**Use of Generative Artificial Intelligence During Manuscript Preparation**

# Overview

This appendix describes the use of generative artificial intelligence (AI) during manuscript preparation and delineates the scope and limitations of its application.

# Generative Artificial Intelligence Tool Used

The authors used Gemini 3.1 Pro (Google DeepMind) during manuscript preparation [1,2].

# Scope of Use

Generative AI was used exclusively for limited editorial support during manuscript development. Specifically, the tool assisted in:

- wording refinement;
- sentence structure organization;
- brainstorming of section structure;
- drafting preliminary English expressions; and
- improving readability and flow.

# Tasks Not Delegated to Generative Artificial Intelligence

Generative AI was not used to:

- make final decisions regarding study design;
- generate or interpret raw study data;
- conduct statistical analyses;
- determine the final scientific interpretation of results;
- decide on the inclusion or exclusion of references; or
- approve the final manuscript content.

# Author Oversight and Accountability

The authors reviewed, edited, and verified all AI-generated outputs. They manually checked factual statements, assessed the relevance and accuracy of references, and revised wording as needed. The authors assume full responsibility for the final content of the manuscript.

# Confidentiality and Responsible Use

The authors did not use generative AI as a substitute for scholarly judgment. They handled sensitive research information with care and did not adopt AI-generated text without critical evaluation. The authors acknowledge that generative AI tools may produce inaccurate statements or fabricated references; therefore, they independently verified all material incorporated into the manuscript [3].

# Record Retention

The authors retained records of prompts and outputs generated during manuscript preparation. These records are available for editorial review upon request.

# References

1. Google Gemini. Meet Gemini, Google’s AI assistant. Google. Accessed April 17, 2026. <https://gemini.google.com/>
2. Google DeepMind. Gemini 3.1 Pro. Google DeepMind. Accessed April 17, 2026. <https://deepmind.google/models/gemini/pro/>
3. Leung T, de Azevedo Cardoso T, Mavragani A, Eysenbach G. Best practices for using AI tools as an author, peer reviewer, or editor. J Med Internet Res. 2023;25:e51584. doi:10.2196/51584
