# Supplementary material for: Feasibility and Preliminary Effects of AI-Generated Personalized Sleep Feedback in High School Female Soccer Players: Pilot Randomized Controlled Trial
Source: JMIR Form Res. 2026 Oct 1;10:e101615. doi: 10.2196/101615 (PMC13673175; doi:10.2196/101615)
Supplement: Supplementary file 4 [file formative_v10i1e101615_app4.docx]

**Multimedia Appendix 4**

**Adaptation and Operational Use of the OSTRC-H-J in This Study**

# Overview

This appendix describes the adaptation and operationalization of the Japanese version of the Oslo Sports Trauma Research Center Questionnaire on Health Problems (OSTRC-H-J) for use in this pilot randomized controlled trial. To avoid reproducing copyrighted questionnaire content, only the adaptation procedures, operational modifications, and scoring approach used in this study are reported.

Purpose of Adaptation
The OSTRC-H-J is the Japanese adaptation of the Oslo Sports Trauma Research Center questionnaire framework for monitoring overuse injuries and health problems in sport [1]. The original OSTRC instrument assesses health problems over the preceding 7 days [2]. In the present study, we operationally modified the instrument to align its recall period with other self-reported measures administered at baseline and postintervention. Accordingly, the recall period was extended from 7 days to 1 month. In addition, the assessment was restricted to sports injuries.

Summary of Modifications
The study-specific adaptations included the following:

- The recall period was extended from 7 days to 1 month.
- The assessment scope was restricted to sports injuries.
- The four conceptual domains were retained: participation, training volume, sports performance, and symptom severity.
- The original weighted severity scoring method was retained.

Operational Interpretation in This Study
The adapted questionnaire assessed the extent to which sports injuries affected:

- participation in training and competition;
- training volume;
- sports performance; and
- symptom severity over the preceding month.

The resulting score was interpreted as an index of injury-related burden. Higher scores indicated a greater impact of sports injuries on athletic participation and performance.

Scoring Procedure
Responses were converted into a 0 to 100 severity score using the weighted method proposed by Clarsen et al [2]. The original weighting structure was retained. Item scores were summed to generate an overall severity score, with higher values indicating greater injury-related burden.

Rationale for Retaining the Scoring System
Although the recall period was extended for operational consistency with other study measures, the conceptual structure and severity scoring framework of the instrument were preserved to maintain interpretability within the OSTRC framework [2,3].

Methodological Note
The updated OSTRC framework emphasizes flexible and improved reporting of sports-related health problems and provides a broader conceptual foundation for instrument use [3]. In this study, the modified recall period was implemented to ensure harmonization of measurement timing across outcomes.

Limitation of Adaptation
This operational modification was implemented to harmonize assessment timing across study measures. However, extending the recall period from 7 days to 1 month may reduce recall accuracy and limit comparability with studies using the standard 7-day format. Accordingly, the modified score was treated as an exploratory outcome in this study.

# References

1. Mashimo S, Yoshida N, Hogan T, Takegami A, Hirono J, Matsuki Y, Hagiwara M, Nagano Y. Japanese translation and validation of web-based questionnaires on overuse injuries and health problems. PLoS One. 2020;15(12):e0242993. doi:10.1371/journal.pone.0242993. PMID:33270675.
2. Clarsen B, Myklebust G, Bahr R. Development and validation of a new method for the registration of overuse injuries in sports injury epidemiology: the Oslo Sports Trauma Research Centre overuse injury questionnaire. Br J Sports Med. 2013;47(8):495-502. doi:10.1136/bjsports-2012-091524. PMID:23038786.
3. Clarsen B, Bahr R, Myklebust G, Andersson SH, Docking SI, Drew M, Finch CF, Fortington LV, Harøy J, Khan KM, et al. Improved reporting of overuse injuries and health problems in sport: an update of the Oslo Sport Trauma Research Center questionnaires. Br J Sports Med. 2020;54(7):390-396. doi:10.1136/bjsports-2019-101337. PMID:32060142.
